# Conditioning in Tropical Probability Theory

**DOI:** 10.3390/e25121641

**Published:** 2023-12-09

**Authors:** Rostislav Matveev, Jacobus W. Portegies

**Affiliations:** 1 Max Planck Institute for Mathematics in the Sciences, 04103 Leipzig, Germany; 2 Department of Mathematics and Computer Science, Eindhoven University of Technology, 5600 MB Eindhoven, The Netherlands

**Keywords:** tropical probability

## Abstract

We define a natural operation of conditioning of tropical diagrams of probability spaces and show that it is Lipschitz continuous with respect to the asymptotic entropy distance.

## 1. Introduction

In [1,2], we have initiated the study of tropical probability spaces and their diagrams. In [1], we endowed (commutative) diagrams of probability spaces with the intrinsic entropy distance and, in [2], we defined tropical diagrams as points in the asymptotic cone of the metric space. They are represented by certain sequences of diagrams of probability spaces.

We expect that tropical diagrams will be helpful in the study of information optimization problems, such as the ones considered in [3,4,5,6,7,8], and we have indeed applied them to derive a dimension-reduction result for the shape of the entropic cone in [9].

In this present article, we introduce the notion of conditioning on a space in a tropical diagram and show that the operation is Lipschitz continuous with respect to the asymptotic entropy distance.

It is a rather technical result, and we have, therefore, decided to treat it in this separate article, but it is an important ingredient in the theory and, in particular, we need it for the dimension-reduction result mentioned before.

Given a tuple of finite-valued random variables ${\left(X_{i}\right)}_{i=1}^{n}$ and a random variable Y, one may "condition" the collection $\left(X_{i}\right)$ on Y. The result of this operation is a family of *n*-tuples of random variables denoted ${\left(X_{i}|Y\right)}_{i=1}^{n}$ parameterized by those values of Y that have positive probability. Each tuple of random variables in this family is defined on a separate probability space.

When passing to the tropical setting, the situation is different in the sense that when we condition a tropical diagram [X] on a space [Y], the result is again a tropical diagram [X|Y] rather than a family. After recalling some preliminaries in Section 2, we describe the operation of conditioning and prove that the result depends in a Lipschitz way on the original diagram in Section 3.

## 2. Preliminaries

Our main objects of study are commutative diagrams of probability spaces and their tropical counterparts. In this section, we recall briefly the main definitions and results.

### 2.1. Probability Spaces and Their Diagrams

#### 2.1.1. Probability Spaces

By a *finite probability space,* we mean a set with a probability measure that has finite support. A *reduction* from one probability space to another is an equivalence class of measure-preserving maps. Two maps are equivalent if they coincide on a set of full measures. We call a point *x* in a probability space $X=(\underline{X},p)$ an *atom* if it has positive weight, and we write x∈X to mean *x* is an atom in *X* (as opposed to $x\in \underline{X}$ for points in the underlying set). For a probability space *X*, we denote by |X| the cardinality of the support of the probability measure.

#### 2.1.2. Indexing Categories

To record the combinatorial structure of commutative diagrams of probability spaces and reductions, we use an object that we call an *indexing category*. By an indexing category, we mean a finite category G such that for any pair of objects i,j∈G, there is at most one morphism between them either way. In addition, we will assume it satisfies one additional property that we will describe after introducing some terminology. For a pair of objects i,j∈G such that there is a morphism $\gamma_{ij}:i\to j$, object *i* will be called an *ancestor* of *j* and object *j* will be called a *descendant* of *i*. The subcategory of all descendants of an object i∈G is called an *ideal* generated by *i* and will be denoted $\left\lceil i\right\rceil$, while we will call the subcategory consisting of all ancestors of *i* together with all the morphisms in it a *co-ideal* generated by *i* and denote it by $\left\lfloor i\right\rfloor$. (The term *filter* is also used for a co-ideal in the literature about lattices).

The additional property that an indexing category has to satisfy is that for any pair of objects i,j∈G, there exists a *minimal common ancestor* $\hat{𝚤}$, and $\hat{𝚤}$ is an ancestor for both *i* and *j* and any other ancestor of them both is also an ancestor of $\hat{𝚤}$; in other words, G is an upper semi-lattice.

An equivalent formulation of the property above is the following: the intersection of the co-ideals generated by two objects i,j∈G is also a co-ideal generated by some object $\hat{𝚤}\in G$.

Any indexing category G is necessarily *initial*, which means that there exists an *initial object*, that is an object $i_{0}$ such that $G=\left\lceil i_{0}\right\rceil$.

A *fan* in a category is a pair of morphisms with the same domain. A fan (i←k→j) is called *minimal* if for any other fan (i←l→j) included in a commutative diagram

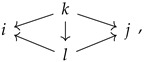

the vertical arrow must be an isomorphism; in other words, *k* is a minimal common ancestor of *i* and *j*.

For any pair of objects i,j in an indexing category G, there exists a unique minimal fan $\left(i\leftarrow \hat{𝚤}\to j\right)$ in G.

#### 2.1.3. Diagrams

We denote by Prob the category of finite probability spaces and reductions, i.e., the equivalence classes of measure-preserving maps. For an indexing category $G=\left\{i;\;\gamma_{ij}\right\}$, a G-diagram is a functor X:G→Prob. A reduction *f* from one G-diagram $X=\left\{X_{i};\;\chi_{ij}\right\}$ to another $Y=\left\{Y_{i};\;\upsilon_{ij}\right\}$ is a natural transformation between the functors. It amounts to a collection of reductions $f_{i}:X_{i}\to Y_{i}$, such that the big diagram consisting of all spaces $X_{i}$, $Y_{i}$ and all morphisms $\chi_{ij}$, $\upsilon_{ij}$ and $f_{i}$ is commutative. The category of G-diagrams and reductions will be denoted as $\mathrm{Prob}\langle G\rangle$. The construction of diagrams could be iterated; thus, we can consider H-diagrams of G-diagrams and denote the corresponding category $\mathrm{Prob}\langle G\rangle \langle H\rangle =\mathrm{Prob}\langle G,H\rangle$. Every H-diagram of a G-diagram can also be considered as G-diagrams of H-diagrams; thus, there is a natural equivalence of categories $\mathrm{Prob}\langle G,H\rangle ≅\mathrm{Prob}\langle H,G\rangle$.

A G-diagram X will be called *minimal* if it maps minimal fans in G to minimal fans in the target category. The subspace of all minimal G-diagrams will be denoted $\mathrm{Prob}{\langle G\rangle }_{m}$. In [1], we have shown that for any fan in Prob or in $\mathrm{Prob}\langle G\rangle$, its minimization exists and is unique up to isomorphism.

#### 2.1.4. Tensor Product

The tensor product of two probability spaces $X=(\underline{X\;}\;,p)$ and $Y=(\underline{Y\;}\;,q)$ is their independent product $X⊗Y:=(\underline{X\;}\;\times \underline{Y\;}\;,p⊗q)$. For two G-diagrams $X=\left\{X_{i};\;\chi_{ij}\right\}$ and $Y=\left\{Y_{i};\;\upsilon_{ij}\right\}$, we define their tensor product to be $X⊗Y=\left\{X_{i}⊗Y;\;\chi_{ij}\times \upsilon_{ij}\right\}$.

#### 2.1.5. Constant Diagrams

Given an indexing category G and a probability space, we can form a *constant* diagram $X^{G}$ that has all spaces equal to *X* and all reductions equal to the identity isomorphism. Sometimes, when such a constant diagram is included in a diagram with other G-diagrams (such as, for example, a reduction $X\to X^{G}$), we will write simply *X* in place of $X^{G}$.

#### 2.1.6. Entropy

Evaluating entropy on every space in a G-diagram, we obtain a tuple of non-negative numbers indexed by objects in G; thus, entropy gives a map

$${\mathrm{Ent}}_{*}:\mathrm{Prob}\langle G\rangle \to R^{G},$$

where the target space $R^{G}$ is a space of real-valued functions on the set of objects in G endowed with the $\ell^{1}$-norm. Entropy is a homomorphism in that it satisfies

$${\mathrm{Ent}}_{*}\left(X⊗Y\right)={\mathrm{Ent}}_{*}\left(X\right)+{\mathrm{Ent}}_{*}\left(Y\right).$$

#### 2.1.7. Entropy Distance

Let G be an indexing category and K=(X←Z→Y) be a fan of G-diagrams. We define the *entropy distance* as

$$\mathrm{kd}\left(K\right):={∥{\mathrm{Ent}}_{*}Z-{\mathrm{Ent}}_{*}X∥}_{1}+{∥{\mathrm{Ent}}_{*}Z-{\mathrm{Ent}}_{*}Y∥}_{1}.$$

The *intrinsic entropy distance* between two G-diagrams is defined to be the infimal entropy distance of all fans with terminal diagrams X and Y:

$$k\left(X,Y\right):=\inf\left\{\mathrm{kd}\left(K\right)\;:\;K=\left(X\leftarrow Z\to Y\right)\right\}.$$

The intrinsic entropy distance was introduced in [10,11] for probability spaces.

In [1], it is shown that the infimum is attained, that the optimal fan is minimal, that k is a pseudo-distance, which vanishes if, and only if, X and Y are isomorphic, and that ${\mathrm{Ent}}_{*}$ is a 1-Lipschitz linear functional with respect to k.

### 2.2. Diagrams of Sets, Distributions, and Empirical Reductions

#### 2.2.1. Distributions on Sets

For a set *S*, we denote by ΔS the collection of all finitely-supported probability distributions on *S*. For a pair of distributions $\pi_{1},\pi_{2}\in \Delta S$, we denote by ${∥\pi_{1}-\pi_{2}∥}_{1}$ the *total variation distance* between them.

For a map $f:S\to S^{\prime }$ between two sets, we denote by $f_{*}:\Delta S\to \Delta S^{\prime }$ the induced affine map (the map-preserving convex combinations).

For n∈N, we define the *empirical map* $q:S^{n}\to \Delta S$ by the assignment below. For $\bar{s}=\left(s_{1},\cdots ,s_{n}\right)\in S^{n}$ and A⊂S, we define

$$q\left(\bar{s}\right)\left(A\right):=\frac{1}{n}\cdot |\;\left\{k\;:\;s_{k}\in A\right\}\;|.$$

For a finite probability space X=(S,p), the *empirical distribution* on ΔX is the push-forward $\tau_{n}:=q_{*}p^{⊗n}$. Thus,

$$q:X^{n}\to \left(\Delta X,\tau_{n}\right)$$

is a reduction of finite probability spaces. The construction of empirical reduction is functorial, which is for a reduction between two probability spaces f:X→Y, the diagram of the reductions

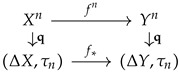
commutes.

#### 2.2.2. Distributions on Diagrams of Sets

Let Set denote the category of sets and surjective maps. For an indexing category G, we denote by $\mathrm{Set}\langle G\rangle$ the category of G-diagrams in Set. The objects in $\mathrm{Set}\langle G\rangle$ are commutative diagrams of sets indexed by G, and the spaces in such a diagram are sets, where the arrows represent surjective maps, subject to commutativity relations.

For a diagram of sets $S=\left\{S_{i};\sigma_{ij}\right\}$, we define the *space of distributions on the diagram* S by

$$\Delta S:=\left\{\left(\pi_{i}\right)\in \prod_{i}\Delta S_{i}\;:\;{\left(\sigma_{ij}\right)}_{*}\pi_{i}=\pi_{j}\right\}.$$

If $S_{0}$ is the initial set of S, then there is an isomorphism of

(1) $$\begin{array}{cc} \Delta S_{0} & \;\overset{≅}{↔}\Delta S\\ \Delta S_{0}∋\pi_{0} & \;\mapsto \left\{{\left(\sigma_{0i}\right)}_{*}\pi_{0}\right\}\in \Delta S\\ \Delta S_{0}∋\pi_{0} & \;↤\left\{\pi_{i}\right\}\in \Delta S. \end{array}$$

Given a G-diagram of sets $S=\left\{S_{i};\sigma_{ij}\right\}$ and an element π∈ΔS, we can construct a G-diagram of probability spaces $\left(S,\pi \right):=\left\{\left(S_{i},\pi_{i}\right);\sigma_{ij}\right\}$. Note that any diagram X of probability spaces has this form.

### 2.3. Conditioning

Consider a G-diagram of probability spaces X=(S,π), where S is a diagram of sets and π∈ΔS. Let $X_{0}=\left(S_{0},\pi_{0}\right)$ be the initial space in X and $U:=X_{i}$ be another space in X. Since $S_{0}$ is initial, there is a map $\sigma_{0,i}:S_{0}\to S_{i}$. Fix an atom u∈U and define the conditioned distribution $\pi_{0}\left(\cdot |u\right)$ on $S_{0}$ as the distribution supported in $\sigma_{0,i}^{-1}\left(u\right)$ and for every $s\in \sigma_{0,i}^{-1}\left(u\right)$ defined by

$$\pi_{0}\left(s|u\right):=\frac{\pi_{0}\left(s\right)}{\pi_{0}\left(\sigma_{0,i}^{-1}\left(u\right)\right)}.$$

Let π(·|u)∈ΔS be the distribution corresponding to $\pi_{0}\left(\cdot |u\right)$ under the isomorphism in (1). We define the *conditioned* G-diagram as X|u:=(S,π(·|u)).

### 2.4. The Slicing Lemma

In [1], we prove the so-called Slicing Lemma that allows us to estimate the intrinsic entropy distance between two diagrams in terms of distances between conditioned diagrams. Among the corollaries of the Slicing Lemma is the following inequality.

**Proposition 1.**  *Let $\left(X\leftarrow \hat{X}\to U^{G}\right)\in \mathrm{Prob}\langle G,\Lambda_{2}\rangle$ be a fan of G-diagrams of probability spaces and $Y\in \mathrm{Prob}\langle G\rangle$ be another diagram. Then,*

$$k\left(X,Y\right)\leq \int_{U}k\left(X|u,Y\right)\;dp\left(u\right)+2\left[\;\left[G\right]\;\right]\cdot \mathrm{Ent}U.$$

The fan in the assumption of the proposition above can often be constructed in the following manner. Suppose X is a G-diagram and $U:=X_{\iota }$ is a space in it for some ι∈G. We can construct a fan $\left(X\overset{f}{\leftarrow }\hat{X}\overset{g}{\to }U^{G}\right)\in \mathrm{Prob}\langle G,\Lambda_{2}\rangle$ by assigning ${\hat{X}}_{i}$ to be the initial space of the (unique) minimal fan in X with terminal spaces $X_{i}$ and *U* and $f_{i}$ and $g_{i}$ to be left and right reductions in that fan for any i∈G.

### 2.5. Tropical Diagrams

A detailed discussion of the topics in this section can be found in [2].

The asymptotic entropy distance between two diagrams of the same combinatorial type is defined by

$$\kappa \left(X,Y\right):=\lim\frac{1}{n}k\left(X^{n},Y^{n}\right).$$

A tropical G-diagram is an equivalence class of certain sequences of G-diagrams of probability spaces. Below, we describe the type of sequences and the equivalence relation.

A function φ:R≥1→R≥0 is called an *admissible function* if φ is non-decreasing and there is a constant $D_{\varphi }$, such that for any t≥1:

$$t\cdot \int_{t}^{\infty }\frac{\varphi (s)}{s^{2}}\;ds\leq D_{\varphi }\cdot \varphi \left(t\right).$$

An example of an admissible function will be $\varphi \left(t\right)=t^{\alpha }$ for α∈[0,1).

A sequence $\bar{X}=\left(X\left(n\right):\;n\in N_{0}\right)$ of diagrams of probability spaces will be called *quasi-linear* with the *defect* bounded by an admissible function φ if for some C>0 and all m,n∈N, it satisfies

$$\kappa \left(X(n+m),X(n)⊗X(m)\right)\leq C\cdot \varphi \left(n+m\right).$$

For example for a diagram X, the sequence $\vec{X}:=\left(X^{n}:\;n\in N_{0}\right)$ is φ-quasi-linear for φ≡0 (and for any admissible φ). Sequences with zero defect are called *linear*, and the space of all linear sequences in $\mathrm{Prob}\langle G\rangle$ is denoted by $L(\mathrm{Prob}\langle G\rangle )$.

The asymptotic entropic distance between two φ-quasi-linear sequences $\bar{X}=\left(X\left(n\right):\;n\in N_{0}\right)$ and $\bar{Y}=\left(Y\left(n\right):\;n\in N_{0}\right)$ is defined to be

$$\kappa \left(\bar{X},\bar{Y}\right):=\lim_{n\to \infty }\frac{1}{n}k\left(X\left(n\right),Y\left(n\right)\right),$$

and the sequences are called *asymptotically equivalent* if $\kappa (\bar{X},\bar{Y})=0$. An equivalence class of a sequence $\bar{X}$ will be denoted as [X], and the totality of all the classes as Prob[G]. We have shown in [2] that the space of equivalence classes of φ-quasi-linear sequences does not depend on the choice of a non-zero admissible function φ.

The sum of two such equivalence classes is defined to be the equivalence class of the sequence obtained by tensor-multiplying representative sequences of the summands term-wise. In addition, there is a doubly transitive action of $R_{\geq 0}$ on Prob[G]. In [2], the following theorem is proven.

**Theorem 1.**  *Let G be an indexing category. Then:*

*1.*  *The space Prob[G] does not depend on the choice of a positive admissible function φ up to isometry.*
*2.*  *The space Prob[G] is metrically complete.*
*3.*  *The map $X\mapsto \vec{X}$ is a κ-κ-isometric embedding. The space of linear sequences, i.e., the image of the map above, is dense in Prob[G].*
*4.*  *There is a distance-preserving homomorphism from Prob[G] into a Banach space B, whose image is a closed convex cone in B.*
*5.*  *The entropy functional*
$$\begin{array}{cc} {\mathrm{Ent}}_{*}:\mathrm{Prob}\left[G\right] & \to R^{G}\\ {}[{\left(X(n)\right)}_{n\in N_{0}}] & \mapsto \lim_{n\to \infty }\frac{1}{n}{\mathrm{Ent}}_{*}X\left(n\right) \end{array}$$
  *is a well-defined 1-Lipschitz linear map.*

### 2.6. Asymptotic Equipartition Property for Diagrams

Among all G-diagrams, there is a special class of maximally symmetric ones. We call such diagrams *homogeneous*; see below for the definition. Homogeneous diagrams come in very handy in many considerations, because their structure is easier to describe than that of general diagrams. We show below that among the tropical diagrams, those that have homogeneous representatives are dense. It means, in particular, that when considering continuous functionals in the space of diagrams, it suffices to only study them in the space of all homogeneous diagrams.

#### 2.6.1. Homogeneous Diagrams

A G-diagram X is called *homogeneous* if the automorphism group Aut(X) acts transitively on every space in X, by which we mean that the action is transitive on the support of the probability measure. Homogeneous probability spaces are isomorphic to uniform spaces. For more complex indexing categories, this simple description is not sufficient.

#### 2.6.2. Tropical Homogeneous Diagrams

The subcategory of all homogeneous G-diagrams will be denoted $\mathrm{Prob}{\langle G\rangle }_{h}$, and we write $\mathrm{Prob}{\langle G\rangle }_{h,m}$ for the category of minimal homogeneous G-diagrams. These spaces are invariant under the tensor product; thus, they are metric Abelian monoids, and the general "tropicalization" described in [2] can be performed. Passing to the tropical limit, we obtain spaces of tropical (minimal) homogeneous diagrams, which we denote by $\mathrm{Prob}{\left[G\right]}_{h}$ and $\mathrm{Prob}{\left[G\right]}_{h,m}$, respectively.

#### 2.6.3. Asymptotic Equipartition Property

For an indexing category G, denote by [[G]] the number of objects in G. In [1], the following theorem is proven.

**Theorem 2.**  *Suppose $X\in \mathrm{Prob}\langle G\rangle$ is a G-diagram of probability spaces for some fixed indexing category G. Then, there exists a sequence $\bar{H}={\left(H_{n}\right)}_{n=0}^{\infty }$ of homogeneous G-diagrams, such that*

(2) $$\frac{1}{n}k\left(X^{n},H_{n}\right)\leq C(|X_{0}\left|,\left[\;\left[G\right]\;\right]\right)\cdot \sqrt{\frac{\ln^{3}n}{n}},$$

*where $C(|X_{0}|,\left[\;\left[G\right]\;\right])$ is a constant only depending on $|X_{0}|$ and [[G]].*

The approximating sequence of homogeneous diagrams is evidently quasi-linear with the defect bounded by the admissible function

$$\varphi \left(t\right):=2C(|X_{0}\left|,\left[\;\left[G\right]\;\right]\right)\cdot t^{3/4}\geq 2C(|X_{0}\left|,\left[\;\left[G\right]\;\right]\right)\cdot t^{1/2}\cdot \ln^{3/2}t.$$

Thus, Theorem 2 above states that $L\left(\mathrm{Prob}\langle G\rangle \right)\subset \mathrm{Prob}{\left[G\right]}_{h}$. On the other hand, we have shown in [2] that the space of linear sequences $L(\mathrm{Prob}\langle G\rangle )$ is dense in Prob[G]. Combining the two statements, we obtain the following theorem.

**Theorem 3.**  *For any indexing category G, the space $\mathrm{Prob}{\left[G\right]}_{h}$ is dense in Prob[G]. Similarly, the space $\mathrm{Prob}{\left[G\right]}_{h,m}$ is dense in $\mathrm{Prob}{\left[G\right]}_{m}$.*

## 3. Conditioning of Tropical Diagrams

### 3.1. Motivation

Let $X\in \mathrm{Prob}\langle G\rangle$ be a G-diagram of probability spaces containing probability space $U=X_{i_{0}}$ indexed by an object $i_{0}\in G$.

Given an atom u∈U, we can define a conditioned diagram X|u. If the diagram X is homogeneous, then the isomorphism class of X|u is independent of *u*, so that (X|u:u∈U) is a constant family. On the other hand, we have shown that the power of any diagram can be approximated by homogeneous diagrams, thus suggesting that in the tropical setting X|U should be a well-defined tropical diagram, rather than a family. Below, we give a definition of the tropical conditioning operation and prove its consistency.

### 3.2. Classical-Tropical Conditioning

Here, we define the operation of conditioning of the classical diagram, such that the result is a tropical diagram. Let X be a G-diagram of probability spaces and *U* be a space in X. We define the conditioning map

$$\left[\cdot \;|\cdot \right]:\mathrm{Prob}\langle G\rangle \to \mathrm{Prob}\left[G\right]$$

by conditioning X by u∈U and averaging the corresponding tropical diagrams:

$$\left[X|U\right]:=\int_{u\in U}\vec{\left(X|u\right)}\;dp_{U}\left(u\right),$$

where $\vec{\left(X|u\right)}$ is the tropical diagram represented by a linear sequence generated by X|u; see Section 2.5. Note that the integral on the right-hand side is just a finite convex combination of tropical diagrams. Expanding all the definitions, we will obtain for [Y]:=[X|U], the representative sequence

$$Y\left(n\right)=\underset{u\in U}{⨂}{\left(X|u\right)}^{\left\lfloor n\cdot p(u)\right\rfloor }.$$

### 3.3. Properties

#### 3.3.1. Conditioning of Homogeneous Diagrams

If the diagram X is *homogeneous*, then for any atom u∈U, with a positive weight,

$$\left[X|U\right]\;\overset{ˇ}{=}\;\vec{\left(X|u\right)}.$$

#### 3.3.2. Entropy

By definition, the conditioned entropy is

$${\mathrm{Ent}}_{*}\left(X|U\right):=\int_{U}{\mathrm{Ent}}_{*}\left(X|u\right)\;dp_{U}\left(u\right).$$

Now that [X|U] is a tropical diagram, the expression ${\mathrm{Ent}}_{*}\left(X|U\right)$ can be interpreted in two, a priori different, ways: by the formula above and as the entropy of the object introduced in the previous subsection. Fortunately, the numeric value of it does not depend on the interpretation since the entropy is a linear functional on Prob[G].

#### 3.3.3. Additivity

If X and Y are two G-diagrams with $U:=X_{\iota }$, $V:=Y_{\iota }$ for some ι∈G, then

[(X⊗Y)|(U⊗V)]=[X|U]+[Y|V].

**Proof.** 
$$\begin{array}{cc} {}[(X⊗ & Y)|\left(U⊗V\right)]=\int_{U⊗V}\vec{\left(X⊗Y)|(u,v\right)}\;dp\left(u\right)\;dp\left(v\right)\\ \; & =\int_{U⊗V}\left(\vec{X|u}+\vec{Y|v}\right)\;dp\left(u\right)\;dp\left(v\right)=\int_{U}\vec{X|u}\;dp\left(u\right)+\int_{V}\vec{Y|v}\;dp\left(v\right)\\ \; & =[X|U]+[Y|V] \end{array}$$

□

#### 3.3.4. Homogeneity

It follows that for any diagram X, a space *U* in X and $n\in N_{0}$ holds

$$\left[X^{n}|U^{n}\right]=n\cdot \left[X|U\right].$$

### 3.4. Continuity and Lipschitz Property

**Proposition 2.**  *Let G be an indexing category, $X,Y\in \mathrm{Prob}\langle G\rangle$ be two G diagrams, and $U:=X_{\iota }$ and $V:=Y_{\iota }$ be two spaces in X and Y, respectively, indexed by some ι∈G. Then,*

$$\kappa \left([X|U],\;[Y|V]\right)\leq \left(2\cdot \left[\;\left[G\right]\;\right]+1\right)\cdot k\left(X,Y\right).$$

Using the homogeneity property of conditioning, Section 3.3.4, we can obtain the following stronger inequality.

**Corollary 1.**  *In the setting of Proposition 2, the following holds:*

$$\kappa \left([X|U],\;[Y|V]\right)\leq \left(2\cdot \left[\;\left[G\right]\;\right]+1\right)\cdot \kappa \left(X,Y\right).$$

Before we prove Proposition 2, we will need some preparatory lemmas.

**Lemma 1.**  *Let A be a G-diagram of probability spaces and E be a space in it. Let $q:E^{n}\to \left(\Delta E,\tau_{n}\right)$ be the empirical reduction. Then, for any n∈N and any $\bar{e},{\bar{e}}^{\prime }\in ,E^{n}$*

$$k(A^{n}|\bar{e},A^{n}|{\bar{e}}^{\prime })\leq n\cdot ∥{\mathrm{Ent}}_{*}{\left(A\right)∥}_{1}\cdot {∥q\left(\bar{e}\right)-q\left({\bar{e}}^{\prime }\right)∥}_{1}.$$

**Proof.**  To prove the lemma, we construct a coupling between $A^{n}|\bar{e}$ and $A^{n}|{\bar{e}}^{\prime }$ in the following manner. Note that there exists a permutation $\sigma \in S_{n}$, such that

$$|\;\left\{i\;:\;e_{i}\neq e_{\sigma i}^{\prime }\right\}\;|=\frac{n}{2}\cdot {∥q\left(\bar{e}\right)-q\left({\bar{e}}^{\prime }\right)∥}_{1}.$$

Let

$$\begin{array}{cc} I & =\left\{i\;:\;e_{i}=e_{\sigma i}^{\prime }\right\}\\ \tilde{I} & =\left\{i\;:\;e_{i}\neq e_{\sigma i}^{\prime }\right\}. \end{array}$$

Using that $|\tilde{I}|=\frac{n}{2}\cdot {∥q\left(\bar{e}\right)-q\left({\bar{e}}^{\prime }\right)∥}_{1}$, we can estimate

$$\begin{array}{cc} k\left(A^{n}\left|\bar{e}\;,\;A^{n}\right|{\bar{e}}^{\prime }\right) & =k\left(\overset{n}{\underset{i=1}{⨂}}\left(A|e_{i}\right)\;,\;\overset{n}{\underset{i=1}{⨂}}\left(A|e_{\sigma i}^{\prime }\right)\right)\\ \; & \leq \sum_{i\in I}\mathrm{kd}(A|e_{i}\overset{=}{⟷}A|e_{\sigma i}^{\prime })\;+\;\sum_{i\in \tilde{I}}\mathrm{kd}(A|e_{i}\overset{⊗}{⟷}A\left|e_{\sigma i}^{\prime }\right)\\ \; & \leq n\cdot ∥{\mathrm{Ent}}_{*}{\left(A\right)∥}_{1}\cdot {∥q\left(\bar{e}\right)-q\left({\bar{e}}^{\prime }\right)∥}_{1}, \end{array}$$

where $A\overset{=}{↔}B$ denotes the isomorphism coupling of two naturally isomorphic diagrams, while $A\overset{⊗}{↔}B$ denotes the “independence” coupling. □

**Lemma 2.**  *Let A be a G-diagram of probability spaces and E be a space in A. Then,*

$$\int_{E^{n}}k\left(A^{n},A^{n}|\bar{e}\right)\;dp\left(\bar{e}\right)\leq 2n\cdot \left[\;\left[G\right]\;\right]\cdot \mathrm{Ent}\left(E\right)+o\left(n\right).$$

**Proof.**  First, we apply Proposition 1 slicing the first argument:

$$\begin{array}{c} \int_{E^{n}}k\left(A^{n},A^{n}|\bar{e}\right)\;dp\left(\bar{e}\right)\\ \;\leq \int_{E^{n}}\int_{E^{n}}k(A^{n}|{\bar{e}}^{\prime },A^{n}\left|\bar{e}\right)\;dp\left({\bar{e}}^{\prime }\right)\;dp\left(\bar{e}\right)+2n\cdot \left[\;\left[G\right]\;\right]\cdot \mathrm{Ent}\left(E\right). \end{array}$$

We will argue now that the double integral on the right-hand side grows sub-linearly with *n*. We estimate the double integral by applying Lemma 1 to the integrand

$$\begin{array}{c} \int_{E^{n}}\int_{E^{n}}k(A^{n}|{\bar{e}}^{\prime },A^{n}\left|\bar{e}\right)\;dp\left({\bar{e}}^{\prime }\right)\;dp\left(\bar{e}\right)\\ \;\leq \int_{E^{n}}\int_{E^{n}}n\cdot \left[\;\left[G\right]\;\right]\cdot |{\mathrm{Ent}}_{*}{\left(A\right)|}_{1}\cdot {\left|q\left(\bar{e}\right)-q\left({\bar{e}}^{\prime }\right)\right|}_{1}\;dp\left({\bar{e}}^{\prime }\right)\;dp\left(\bar{e}\right)\\ \;=n\cdot \left[\;\left[G\right]\;\right]\cdot |{\mathrm{Ent}}_{*}{\left(A\right)|}_{1}\cdot \int_{\Delta E}\int_{\Delta E}{\left|\pi -\pi^{\prime }\right|}_{1}\;d\tau_{n}\left(\pi \right)\;d\tau_{n}\left(\pi^{\prime }\right)=o\left(n\right), \end{array}$$

where the convergence to zero of the last double integral follows from Sanov’s theorem. □

**Corollary 2.**  *Let A be a G-diagram and E a probability space included in A. Then,*

$$\kappa \left(\vec{A},\left[A|E\right]\right)\leq 2\left[\;\left[G\right]\;\right]\cdot \mathrm{Ent}\left(E\right).$$

**Proof.**  Let n∈N. Then,

$$\begin{array}{cc} \kappa \left(\vec{A},\left[A|E\right]\right)\; & =\frac{1}{n}\kappa \left(\vec{A^{n}},\left[A^{n}|E^{n}\right]\right)\\ \; & =\frac{1}{n}\kappa \left(\vec{A^{n}},\int_{E^{n}}\vec{A^{n}|\bar{e}}\;dp\left(\bar{e}\right)\right)\\ \; & \leq \frac{1}{n}\int_{E^{n}}\kappa \left(\vec{A^{n}},\;\vec{A^{n}|\bar{e}}\right)\;dp\left(\bar{e}\right)\\ \; & =\frac{1}{n}\int_{E^{n}}\kappa \left(A^{n},A^{n}|\bar{e}\right)\;dp\left(\bar{e}\right)\\ \; & \leq 2\cdot \left[\;\left[G\right]\;\right]\cdot \mathrm{Ent}\left(E\right)+o\left(n^{0}\right), \end{array}$$

where we used Lemma 2 and the fact that κ≤k in the last line. We finish the proof by taking the limit n→∞. □

**Proof** **of Proposition 2.** We start with a note on general terminology: a reduction f:A→B of probability spaces can also be considered as a fan $F:=(A\overset{=}{\leftarrow }A\overset{f}{\to }B)$. Then, the entropy distance of *f* is

kd(f):=kd(F)=EntA−EntB.

If the reduction *f* is a part of a bigger diagram containing also space *U*, then the following inequality holds:

$$\int_{U}\mathrm{kd}\left(f|u\right)\;dp\left(u\right)\leq \mathrm{kd}\left(f\right).$$

Let $K\in \mathrm{Prob}\langle G,\Lambda_{2}\rangle$

$$K=(X\overset{f}{\leftarrow }Z\overset{g}{\to }Y)\in \mathrm{Prob}\langle G,\Lambda_{2}\rangle =\mathrm{Prob}\langle \Lambda_{2},G\rangle$$

be an optimal coupling between X and Y. It can also be viewed as a G-diagram of two fans, $K={\left\{K_{i}\right\}}_{i\in G}$, each of which is a minimal coupling between $X_{i}$ and $Y_{i}$. Among them is the minimal fan $W:=K_{\iota }=\left(U\overset{f_{\iota }}{⟵}W\overset{g_{\iota }}{⟶}V\right)$.

We use the triangle inequality to bound the distance $\kappa \left([X|U],[Y|V]\right)$ by four summands as follows:

$$\begin{array}{cc} \kappa \left([X|U],[Y|V]\right)\leq & \;\kappa \left([X|U],[Z|U]\right)\;+\kappa \left([Z|U],[Z|W]\right)\;+\\ & \;\kappa \left([Z|W],[Z|V]\right)+\kappa \left([Z|V],[Y|V]\right). \end{array}$$

We will estimate each of the four summands separately. The bound for the first one is as follows:

$$\begin{array}{c} \kappa (\left[X|U\right],\left[Z|U\right])=\kappa \left(\int_{U}\vec{X|u}\;dp\left(u\right),\int_{U}\vec{Z|u}\;dp\left(u\right)\right)\\ \;\leq \int_{U}\kappa \left(\vec{X|u},\;\vec{Z|u}\right)\;dp\left(u\right)=\int_{U}\kappa \left(X|u,\;Z|u\right)\;dp\left(u\right)\\ \;\leq \int_{U}k\left(X|u,\;Z|u\right)\;dp\left(u\right)\leq \int_{U}\mathrm{kd}\left(f|u\right)\;dp\left(u\right)\\ \;\leq \sum_{i\in G}\int_{U}\mathrm{kd}\left(f_{i}|u\right)\;dp\left(u\right)=\sum_{i\in G}\mathrm{kd}\left(f_{i}\right)=\mathrm{kd}\left(f\right). \end{array}$$

An analogous calculation shows that

$$\kappa \left([Z|V],[Y|V]\right)\leq \mathrm{kd}\left(g\right).$$

To bound the second summand, we will use Corollary 2:

$$\begin{array}{cc} \kappa \left([Z|U],[Z|W]\right)\; & =\kappa \left(\int_{U}\vec{Z|u}\;dp\left(u\right),\int_{W}\vec{Z|w}\;dp\left(w\right)\right)\\ \; & =\kappa \left(\int_{U}\vec{Z|u}\;dp\left(u\right),\int_{U}\int_{W|u}\vec{Z|w}\;dp\left(w|u\right)\;dp\left(u\right)\right)\\ \; & \leq \int_{U}\kappa \left(\vec{Z|u},\int_{W|u}\vec{Z|w}\;dp\left(w|u\right)\right)\;dp\left(u\right). \end{array}$$

We will now use Corollary 2 with A=Z|u and E=W|u to estimate the integrand. Then,

$$\begin{array}{cc} \kappa \left([Z|U],[Z|W]\right)\; & =\int_{U}\kappa \left(\vec{Z|u},\int_{W|u}\vec{Z|w}\;dp\left(w|u\right)\right)\;dp\left(u\right)\\ \; & \leq 2\left[\;\left[G\right]\;\right]\cdot \int_{U}\mathrm{Ent}\left(W|u\right)\;dp\left(u\right)\\ \; & \leq 2[\;[G]\;]\cdot \mathrm{Ent}(W|U)\leq 2[\;[G]\;]\cdot \mathrm{kd}(f). \end{array}$$

Similarly,

$$\kappa \left([Z|W],[Z|V]\right)\leq 2\left[\;\left[G\right]\;\right]\cdot \mathrm{kd}\left(g\right).$$

Combining the estimates, we obtain

$$\kappa \left([X|U],[Y|V]\right)\leq \left(2\left[\;\left[G\right]\;\right]+1\right)\cdot \left(\mathrm{kd}\left(f\right)+\mathrm{kd}\left(g\right)\right)=\left(2\left[\;\left[G\right]\;\right]+1\right)\cdot k\left(X,Y\right).$$

□

### 3.5. Tropical Conditioning

Let [X] be a tropical G-diagram and $\left[U\right]=\left[X_{\iota }\right]$ for some ι∈G. Choose a representative ${\left(X(n)\right)}_{n\in N_{0}}$ and denote $u\left(n\right):=X_{\iota }\left(n\right)$. We define now a conditioned diagram [X|U] by the following limit:

$$\left[X|U\right]:=\lim_{n\to \infty }\frac{1}{n}\left[X\left(n\right)|U\left(n\right)\right].$$

Proposition 1 guarantees that the limit exists and is independent of the choice of representative. For a fixed ι∈G, the conditioning is a linear Lipschitz map of

$$\left[\;\cdot \;|\;\cdot_{\iota }\;\right]:\mathrm{Prob}\left[G\right]\to \mathrm{Prob}\left[G\right].$$

## Data Availability

No new data were created or analyzed in this study. Data sharing is not applicable to this article.
